# Relationship between health spending, life expectancy and renewable energy in China: A new evidence from the VECM approach

**DOI:** 10.3389/fpubh.2022.993546

**Published:** 2022-10-20

**Authors:** Hui Liu, Kaiyang Zhong

**Affiliations:** ^1^School of Finance and Taxation, Shandong University of Finance and Economics, Jinan, China; ^2^School of Economics and Management, Binzhou University, Binzhou, China; ^3^School of Economic Information Engineering, Southwestern University of Finance and Economics, Chengdu, China

**Keywords:** health spending, China, VECM approach, life expectancy, renewable energy

## Abstract

There has been a growing trend in health spending and renewable energy consumption in China over the past few decades, which has positive implications for health outcomes, such as life expectancy. Therefore, the main objective of this study is to empirically analyze the impact of health expenditures and renewable energy on life expectancy in China. We used the time series data from 2000Q1 to 2020Q4 and applied the VECM approach for the data analysis. The results of this study suggest a long run association between health spending, life expectancy and renewable energy. The increase in health spending improves life expectancy, while renewable energy consumption also positively affects life expectancy in China. Therefore, the government should allocate sufficient funding to the health sector in order to attain higher life expectancy in the country. In addition, the government should also provide incentives for the consumption and production of renewable energy, which could help to achieve the sustainable development goal and life expectancy.

## Introduction

Health spending and health outcomes have been extensively discussed in the literature. However, less attention is given to essential factors such as renewable energy. Academicians, policymakers, and the general public have devoted considerable attention to rising healthcare expenditures (1). Health spending may affect the productivity of labor, and the traditional endogenous growth theories assume that human capital accumulation could significantly improve the long run output per worker. People in good health are more productive, and higher public health spending is associated with improved health outcomes, such as high life expectancy (2, 3). Most of the studies related to health outcomes and health spending followed by the production function using health care spending as the explanatory variable while health outcomes such as mortality and life expectancy are taken as dependent variables (4). Health spending may improve life expectancy in the long run and could lead to higher earnings in short run (5). Akinci et al. (6) reported that in Middle East and North Africa (MENA) region, efficient healthcare spending increases life expectancy. Countries with the best health outcomes allocate a large portion of their budgets to healthcare spending (7). Sufficient health spending improves the outcomes. However, insufficient spending on healthcare may create impediments for the poor public to get basic health care facilities.

The economic development in China's post industrialization has increased the energy demand, which has serious concern for public health and sustainable development. Conventional fossil fuel energy could lead to environmental degradation and have negative implications for life expectancy (8). The UN Environment Conference reported that seven million people worldwide died from air pollution each year (9). The energy produced from fossil fuels may produce harmful emissions, for example, carbon monoxide (CO) and carbon dioxide (CO_2_). The energy production from fuel leads to air pollution and increases CO_2_ emissions, which affect environmental quality. Consequently, it leads to global warming, and many living species may become extinct. Increasing global temperatures can also have a negative effect on people since it increases the chance of certain diseases, such as cancer and mental illness, which leads to a shorter life expectancy (10). Therefore, it is essential to switch from non-renewable energy sources to renewable energy sources such as solar and biomass in order to reduce overall environmental degradation, which expectedly, to improve life expectancy. Although human health is associated with many factors that have been discussed in the literature, however, less attention is given to the relationship between renewable energy and life expectancy (11). China made socioeconomic improvements by investing in social sectors such as health, education, environmental management, sanitation, and sustainability. China's rising per capita income and industrialization has led to rise the healthcare expenditures. Over the past few decades, there has been a significant reduction in the prevalence of poverty, as well as improvements in adult literacy, access to safe drinking water, nutrition and nutrition, all of which are likely to contribute in the increase of life expectancy (12). Government spending on healthcare has an increasing trend in China, and globalization increases the real income of the people, which raises energy consumption.

Renewable energy consumption has significant implications for the environment and human health (13, 14). Renewable energy provides clean energy and a clean environment, which could improve human health and life expectancy. Both public health spending and renewable energy consumption could prolong the life expectancy in China. Furthermore, less attention has been given to the renewable energy and life expectancy relationship in literature. Therefore, to fill this research gap, this paper explores the linkages between health spending, renewable consumption and life expectancy in China. This paper contributes to the literature from the following aspects: First, we attempt to link life expectancy with renewable energy and health in China; Second, we use the cointegration methodology to explore long run association between life expectancy, renewable energy, and health spending. Third, we use the case of China, which is an emerging developing country. The recent economic development in the post trade liberation brings a significant improvement in people's income and quality of life in China. Therefore, this study considers the post trade and industrial development period for analysis, which provides a clear policy direction and implications of recent economic development in China for life expectancy. The rest of the paper is organized as follows: Section Health spending, life expectancy, and renewable energy consumption in China shows development facts of the health spending, renewable energy and life expectancy in China; Section Literature review gives the literature review; Section Methodology and Results and discussion show the methodology and results; Section Conclusion shows the conclusion of the study.

## Health spending, life expectancy, and renewable energy consumption in China

**Figure 1:**
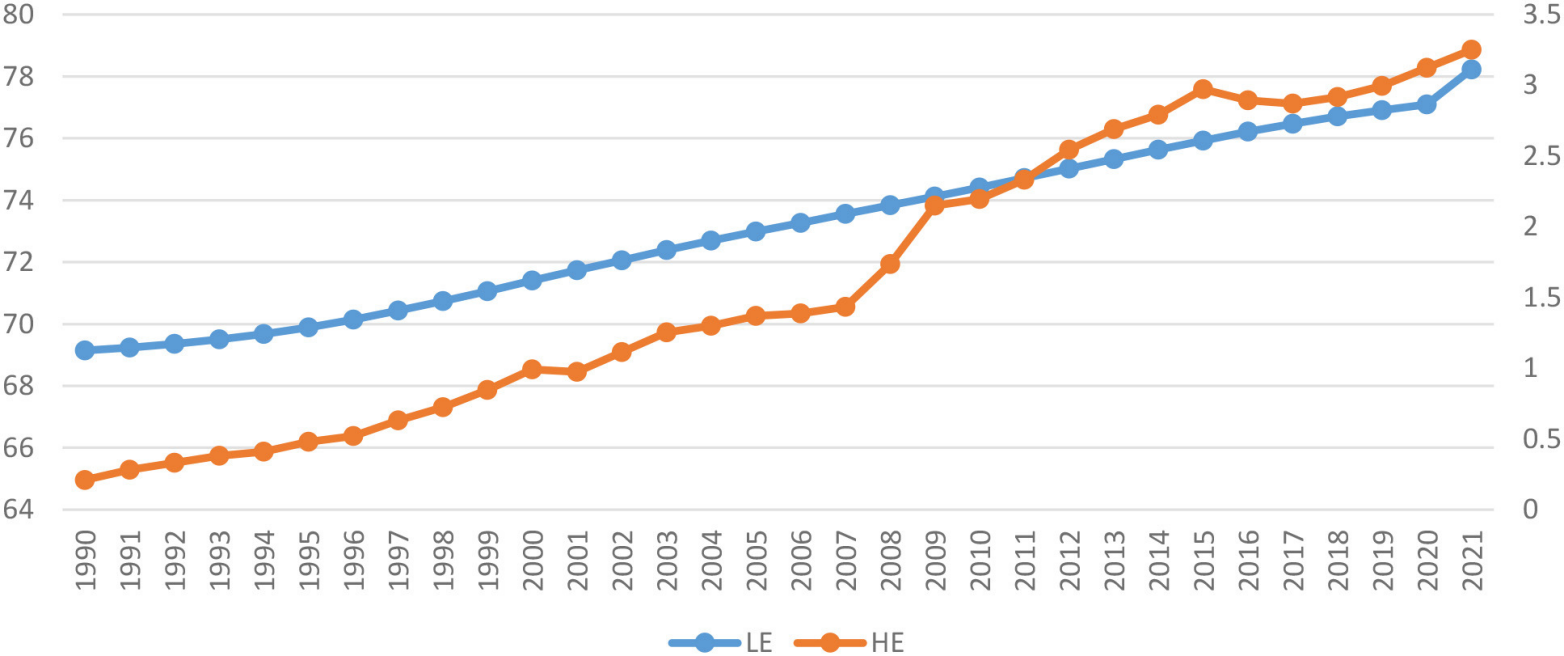
Life expectancy (LE) and Health spending (HE) in China (1990–2021). Source: World Bank Development Indicators.

This section provides information related to renewable health spending in China. Various health reforms have been introduced in China to promote public health. China initiated a comprehensive health-care reform in 2009 and committed to providing all residents equal access to basic health care facilities. The reform in the first phase was focused on increasing social health care coverage for the public and upgrading the infrastructure of the health care system. In the second phase, government reforms mainly focused on the delivery of health, such as the implementation of systemic reform of government hospitals by reducing mark-up for medicine sales and changing fee schedules, and fee for the patients in hospitals (15). In the past few decades, China has made significant progress in expanding equal access to healthcare and improved financial protection, particularly for those with a lower economic class. However, there are still concerns about the quality of care, the prevention and treatment of non-communicable diseases (NCDs), the effectiveness of health care delivery, the management of health care costs, and the level of public satisfaction. Health spending has a continual increasing trend in China from 1990 to 2000, while there is a moderate decrease in health spending from 2000 to 2001. The increasing trend continues from 2001 to 2021 (except for 2016 and 2017). The increasing trend of health spending indicates that the GDP of China is significantly growing over period and leads to an increase in health spending. The overall expenditure on healthcare per capita in China increased from 319 yuan in 2000 to 1888 yuan in 2011 and its representing an average yearly growth rate of 17.4 percent. Between 2000 and 2011, government and social health expenditures per capita rose fast, with an average yearly growth of 22.9 percent, from 56 yuan to 554 yuan, and with an average yearly growth of 18.8 percent, from 92 yuan to 625 yuan, respectively (16). The life expectancy also increased from 1990 to 2021, which implies that due to high spending and steady GDP growth boosted health quality and increased the life expectancy in China. The development of life expectancy and health spending can be seen in Figure 1.

**Figure 2:**
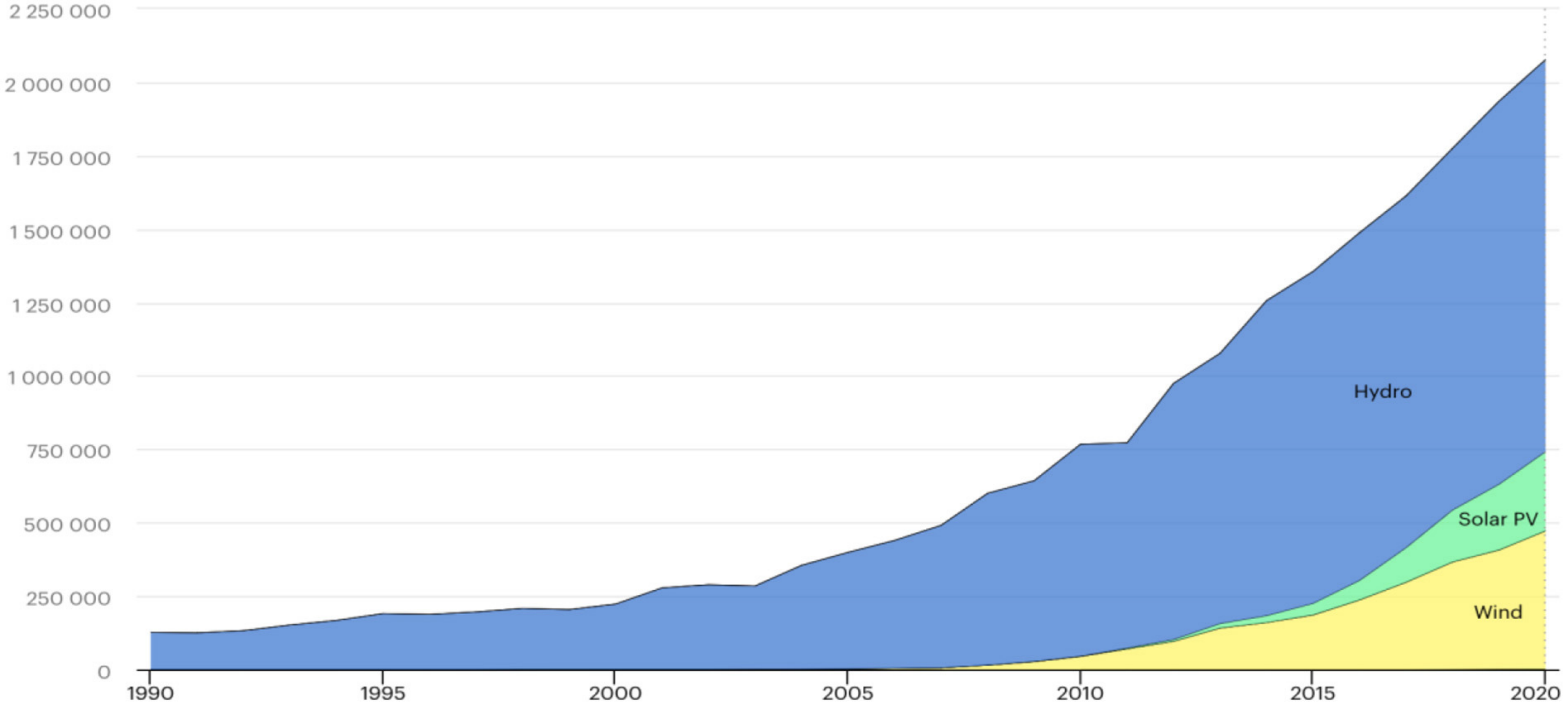
Renewable energy production. Source: (17).

China has been rapidly moving toward a more sustainable and low-carbon future in the past few decades. Carbon-based fuels are gradually being replaced with renewable sources, and clean air laws contribute to a healthy environment and sustainable development in both direct and indirect ways. China already leads in terms of renewable energy output, and becomes the world's largest producer of solar and wind energy, as well as the leading domestic and foreign investor in renewable energy. In 2016, Chinese corporations signed four of the world's top five renewable energy agreements. Investments in renewable energy is the top priority by the Chinese government because they can help the country overcome environmental and socioeconomic challenges. To reduce overall air pollution, the Chinese government actively promotes renewable energy use. According to the National People's Congress (NPC) Environmental Committee report, the production and consumption of energy produced from fossil fuels are responsible for 90 percent of sulfur dioxide emissions in China. The effect of air pollution on health and economy has been extensively studied. RAND Corporation estimates that in 2012, labor productivity losses caused by air pollution cost $535 billion, or 6.5 percent of its GDP in China. Currently, China is actively involved in producing green energy, and its 13th Five Year Plan aims to reduce fossil fuel energy usage and increase the production of renewable energy from 35 to 39% by 2020. China is expected to reach one fifth of its electricity consumption from non-fossil fuel sources by 2030. The International Energy Agency reports that over the next 5 years, China will produce 36% and 40% of the world's solar and wind energy, respectively. Figure 2 represents renewable energy generation in China from 1990 to 2021. Hydro energy is a major source of renewable energy production, in the year 1990 Hydro produced 54.0 GWh, which continuously increased. The second main contributor is the Wind energy; which grew drastically in 2005 and in 2020 wind energy produced 471,175 GWh. Solar energy comes as the third larger contributor to renewable energy, and there is also an increasing trend; however, solar energy started a significant contribution in 2005 and onward. In 1990 it only produced 2.0 GWh, which grew significantly, and it reached 269,718 GWh in 2020.

**Figure 3:**
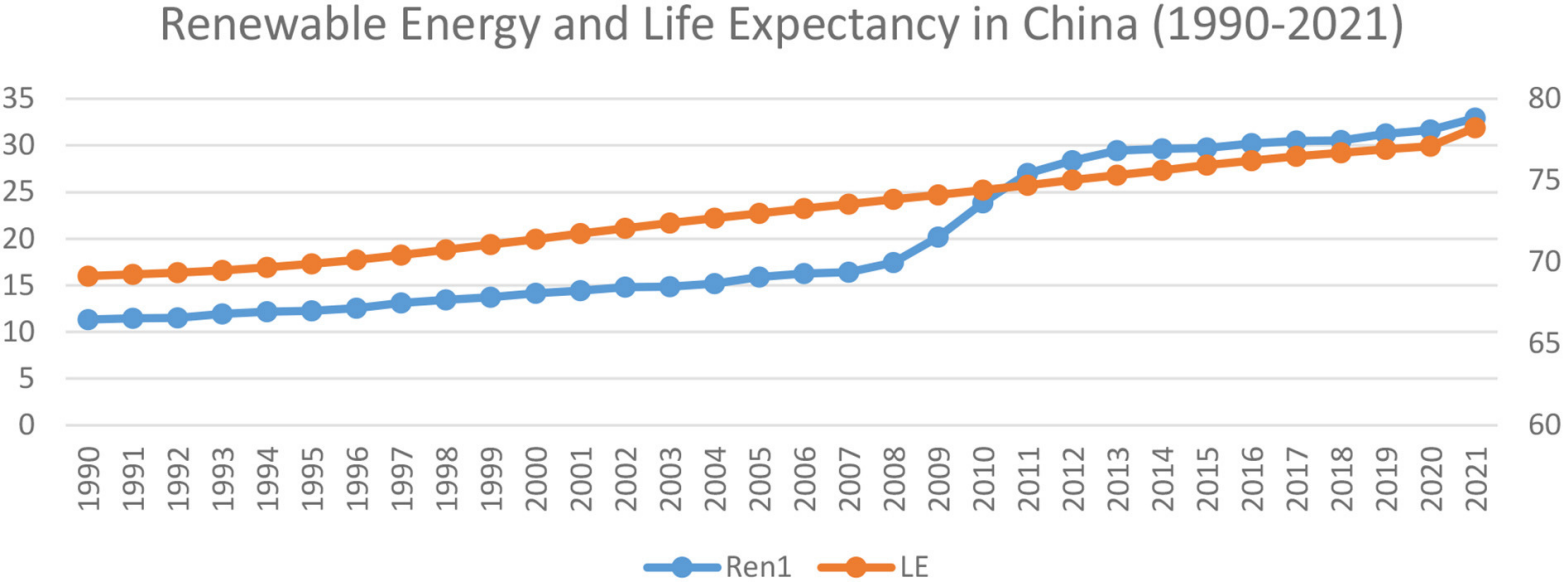
Life expectancy and renewable energy consumption in China (1990–2021).

Figure 3 shows the trend between life expectancy and renewable energy; overall, there is a close association between life expectancy and renewable energy consumption. This implies that, along with other factors, renewable energy is the main factor responsible for the life expectancy in China. According to a study conducted at UC Berkeley, fossil fuel energy has serious health concerns. As a result, air pollution accounts for around 17 percent of all deaths in China. A University of Chicago study estimated that suspended particles in northern China cause half a billion people to lose 5 years of their life expectancy. The Chinese government ranked air pollution as their top priority, and air pollution is the second biggest concern for Chinese citizens. Nevertheless, several participants have less hope, and many participants think air pollution will worsen over the next 5 years. The renewable energy source may become a prominent source of energy in the future that will contribute to sustainable development and the improvement of life expectancy.

## Literature review

In this section we review the literature, which discusses the relationship between health expenditures and health outcomes, e.g., life expectancy, but less attention has been given to the relationship between renewable energy and life expectancy. Lubitz et al. (18) investigated the implications of health spending for elderly people. The elderly with good health live longer as compared to those who did not have good health, assuming the accumulative health spending was the same for both category people. It was possible to improve the health and life expectancy of the elderly without increasing health care spending if promotion efforts were directed toward those under the age of 65. Zeeshan et al. (19) explored the asymmetric relationship between CO_2_ emissions, environmental pollution, and household-level health spending using the case of China. Their results showed an asymmetric relationship between CO_2_ emissions and health spending, argued that an increase in environmental pollution could raise health spending. Owumi and Eboh (20) studied the health spending on life expectancy in Nigeria. They found that life expectancy improved significantly as a result of domestic and external health expenditures during the period of 2000–2017. In addition, government spending could improve life expectancy, and results reported that 1% increase in Nigeria's general government health expenditure would improve life expectancy by 6%. An increase in out-of-pocket health care expenses of 1% would result in improving life expectancy by 63%. In addition, a 1% increase in external health spending would rise in life expectancy by 11%. Shahbaz et al. (21) analyzed the determinants of life expectancy under the role of economic misery using the case of Pakistan. Their results showed that, among the other factors, health spending improved life expectancy. The study of Van den Heuvel and Olaroiu (22) explored the relationship between life expectancy and health spending using the case of European countries. They used cross-sectional data for the 31 European countries. They found that health spending was not the main factor affecting life expectancy, but rather social protection spending could improve life expectancy. The results showed that high spending on social protection could increase life expectancy in Europe. Kabir (12) studied determinants of life expectancy in developing countries; the results indicate that among the different factors such as per capita income, education and safe water, however, health spending is the main factor. Jakovljevic et al. (23) examined long run association between health spending and life expectancy in Eastern Europe. The outcomes suggested a positive relationship between life expectancy and health spending in EU members. Anderson and Poullier (24) studied health spending, access, and outcomes trends in industrialized countries. Their finding reported that health spending significantly increased in industrialized countries. Giang (25) analyzed the relationship between life expectancy and private health spending for EU-15. They applied multiple regression analysis for data and their results indicated a positive association between private health expenditures and life expectancy. They also mentioned that GDP per capita and employment also determined health expectancy.

Less attention has been given to explore the relationship between life expectancy and renewable energy in the literature. Caruso et al. (11) studied the relationship between renewable energy consumption, social factors, and health in Europe. They applied Panel Vector Auto Regression (PVAR) technique for the data analysis. Some factors such as modernization and industrialization significantly affected human health in Europe. In contrast, environmental degradation induced by fossil energy might have a negative effect on human health. Fossil fuel energy could be replaced with renewable energy. Their findings showed the growth of renewable energy use and its impact on other social aspects, rather than the existence of causal linkages between health and renewable energy. Shah et al. (26) analyzed the FDI inflow, mortality and renewable energy consumption in China for the period 1998–2020. They reported that a significant amount of FDI inflow had occurred in the last few decades. This FDI inflow improved the economic growth and development in the country, but on the other hand, it brought environmental degradation in terms of higher CO_2_ emissions. They applied nonlinear ARDL approach and used the data for the period 1998–2020. Their findings showed a significant association between renewable energy, mortality and FDI in China. In addition, they found that renewable energy reduces mortality in China. Ibrahim et al. (27) analyzed the non-renewable energy consumption on quality of life for sub-Saharan African countries. Their findings showed that an increase in non-renewable energy reduced life expectancy in sub-Saharan African countries. Shah et al. (8) studied the linkages between trade, CO_2_ emissions and life expectancy in China. Their findings reported that trade increased CO_2_ emissions, which reduced life expectancy in China. They suggested that renewable energy may provide a clean environment and improve life expectancy. Some literature such as Cheng et al. (28) discussed health spending and health outcomes with different variables, including fiscal decentralization, trade and FDI. However, renewable energy and health association has been analyzed little in the literature. Therefore, this research contributes to the literature by adding the renewable energy implication for life expectancy, with the case of China.

## Methodology

### Model

We use the following model for the data analysis


(1) $$\begin{array}{l} LEt=\beta_{0+}\beta_{1}HEt+\beta_{2}GDPt+\beta_{3}Popt+\beta_{3}RENt+\varepsilon t \end{array}$$

Where LE shows the life expectancy, HE represents the health spending, GDP is the per capita income, Pop is the population, and REN shows the renewable energy consumption. The β_1_, β_2_, β_3_… β_*n*_represent the coefficients of the relevant variables and ε is the error term, which expected to be uncorrelated with the variable. Life expectancy determines by several factors such as per capita income (GDP), health spending (HE) and Renewable energy (REN). Therefore, we use life expectancy as dependent variable, while GDP, HE and REN are taken as explanatory variables. The population used as control variable in the model. The data for the relevant variables are obtained from the World Bank Development, the data is available in annual frequency, which has been converted into Quarter frequency using the Eviews software.

### Methodology

The study adopts the Vector Error Correction Model, which assumes that all variables have same order of integration. The estimation of VECM performs under VAR framework, VAR analyzes the interrelationship between included variables in the system by taking lag values of the variables. Since we are using the long run time series data, it's essential to check the unit root properties of the variables. Unit root tests are usually used to find out if the variables are stationary, which could lead to spurious results. In time series, if the variance and mean of a variable remain constant over time, the series is referred to as stationary (i.e., it is not a random walk and does not have a unit root). Alternatively, the series is defined as non-stationary, which means that it has a unit root. There are different techniques used to test the unit root, Augment Ducky Fuller (AFD) and Ducky Fuller (DF) methods are used to estimate the stationary status of the variable. The Augumnetd Dickey and Fuller (ADF) (29) test of unit root is a modified form of Ducky Fuller (DF)(30). Following regression equations are used to test the unit root


(2) $$\begin{array}{lll} Y_{\text{t}} & = & \gamma Y_{\text{t}-1}+\varepsilon_{\text{t}} \end{array}$$

(3) $$\begin{array}{lll} Y_{\text{t}} & = & z_{0}+\gamma Y_{\text{t}-1}+\varepsilon_{\text{t}} \end{array}$$

(4) $$\begin{array}{lll} Y_{\text{t}} & = & z_{0}+\eta t+\gamma Y_{\text{t}-1}+\varepsilon_{\text{t}} \end{array}$$

These equations are estimated for the parameter value of γ, Yt is considered to be unit root or nonstationary. Dickey and Fuller (30) estimated H0: 0 by using the (trace) statistics critical value for hypothesis testing. In addition, error term in Dicky Fuller (DF) is assumed to be uncorrelated; however, if this assumption does hold and the error term is autocorrelated, then Dicky Fuller (DF) will be invalid and we may use Augmented Dicky Fuller (ADF) test, which augments the above equation by including the dependent variable's lag value. Furthermore, ADF test holds that Y is subject to an AR (n) process and assigned a (n)lag difference value of the response variable.


(5) $$\begin{array}{l} Y_{\text{t}}=z_{0}+\eta t+\gamma Y_{\text{t}-1}+\overset{p}{\underset{i}{\sum }}\alpha_{i}{\Delta Y}_{t-i}\;+\varepsilon_{\text{t}} \end{array}$$

In the ADF unit root test, parameter Y_t − 1_ is tested by using Mackinnon's critical and t(tau) statistics values, if it becomes zero, it indicates the presence of unit root, indicating nonstationary in variable, and vice versa. Following to test the order of integration, we proceed to test the evidence of a long run relationship. The key requirement for testing Johansen cointegration is that all variables have integrated at the initial difference I(1). There are several ways for cointegration, including Engle and Granger (31) unit root test and Johansen (32, 33). However, Johansen cointegration provides several advantages as compared to Engel and Granger because Engel and Granger's method does not specify the number of vector(s), but Johansen tests provide information about the numbers of cointegration vectors. The Johansen test is calculated as follows:


(6) $$\begin{array}{l} \Delta Y_{t}=\overset{p-1}{\underset{i}{\sum }}\Gamma_{i}{\Delta Y}_{\text{t}-\text{i}}\;+\Pi Y_{\text{t}-1}+\varepsilon_{\text{t}} \end{array}$$

In Johansen techniques, the error term is required to estimate likelihood ratios (LR) to determine the cointegrating vectors of Yt in the system. The first test is based on the hypothesis that the rank is equal to or smaller than the cointegrating vector (r), whereas the second is based on trace statistics.


(7) $$\begin{array}{l} \lambda trace=-T\overset{n}{\underset{i=r+1}{\sum }}\text{In}\left(1\;{\text{λ}}_{\text{i}}\right) \end{array}$$

The second test in cointegration provides a maximal Eigen value that tests the null hypothesis for the number of cointegrating vectors as follows:


(8) $$\begin{array}{l} \lambda max=-TIn\left(1-\lambda_{r}\right) \end{array}$$

Because the cointegration test is performed in a VAR setting, we use the Akike and Shward criterion to estimate the suitable lag length. If cointegration is detected, we can proceed to identify the short run deviation from the long run equation known as Error correction model(ECM) for the vectors.


(9) $$\begin{array}{lll} \Delta LE & = & \;\overset{k}{\underset{i=1}{\sum }}\beta_{1}HE_{t-i}+\;\overset{k}{\underset{i=1}{\sum }}\beta_{2}GDP_{t-i} \end{array}$$

$$\begin{array}{ll} + & \overset{k}{\underset{i=1}{\sum }}\beta_{3}Pop_{t-i}+\overset{k}{\underset{i=1}{\sum }}\beta_{4}REN_{t-i}+ECM_{1,t-1} \end{array}$$

(10) $$\begin{array}{lll} \Delta HE & = & \;\overset{k}{\underset{i=1}{\sum }}\beta_{5}LE_{t-i}+\;\overset{k}{\underset{i=1}{\sum }}\beta_{6}GDP_{t-i} \end{array}$$

$$\begin{array}{ll} + & \overset{k}{\underset{i=1}{\sum }}\beta_{7}Pop_{t-i}+\overset{k}{\underset{i=1}{\sum }}\beta_{8}REN_{t-i}+ECM_{2,t-1} \end{array}$$

(11) $$\begin{array}{lll} \Delta GDP & = & \overset{k}{\underset{i=1}{\sum }}\beta_{9}HE_{t-i}+\;\overset{k}{\underset{i=1}{\sum }}\beta_{10}LE_{t-i} \end{array}$$

$$\begin{array}{ll} + & \overset{k}{\underset{i=1}{\sum }}\beta_{11}Pop_{t-i}+\overset{k}{\underset{i=1}{\sum }}\beta_{12}REN_{t-i}+ECM_{3,t-1} \end{array}$$

(12) $$\begin{array}{lll} \Delta Pop & = & \overset{k}{\underset{i=1}{\sum }}\beta_{13}HE_{t-i}+\;\overset{k}{\underset{i=1}{\sum }}\beta_{14}GDP_{t-i} \end{array}$$

$$\begin{array}{ll} + & \overset{k}{\underset{i=1}{\sum }}\beta_{15}LE_{t-i}+\overset{k}{\underset{i=1}{\sum }}\beta_{16}REN_{t-i}+ECM_{4,t-1} \end{array}$$

β_1_,β_2_,β_3_….β_*n*_ shows the short run coefficient of the variables included in the model, ECM lag presents the long causality. The short run causality can be estimated by imposing restrictions on the short run parameters under the Wald Test. In addition, we will use impulse response function and variance decomposition analysis to see the variations of included variables.

## Results and discussion

This section provides the results and discussion of this paper. Since we're using long-term time series data, we are estimating the long association between the variables, therefore we used the Vector Error Correction Model (VECM) for our long-term analysis. Cointegration is a prerequisite for the VECM application, but before estimating the cointegration, we must analyze the order of integration. The ADF unit root test is applied for the order of integration. The ADF results are shown in Table 1.

**Table 1:** ADF unit root test.

**Variable**	**At level**	**At first difference**	**Conclusion**	**Order**
LE	−0.8187	−2.6533***	Non stationary at level; Stationary at first difference	I(1)
HE	−0.9216	−3.8109 ***	Non stationary at level; Stationary at first difference	I(1)
GDP	−0.3581	−6.0653 ***	Non stationary at level; Stationary at first difference	I(1)
Pop	0.2202	−2.1069***	Non stationary at level; Stationary at first difference	I(1)
REN	2.2941	−5.4421***	Non stationary at level; Stationary at first difference	I(1)

***Significant at the 1% level.

Table 1 shows the ADF estimations both at first level and at first difference. The outcomes indicate that all variables are non-integration at the first level, while after taking the first difference, it becomes stationary. This implies that all variables have the same order of integration; this fulfills the essential requirement of cointegration. The cointegration requires the selection of optimal lag length; we select the Schwarz information criterion (SC) and Akaike information criterion (AIC) to choose the optimal lag length. In Tables 2 and 3, the trace statistics and maximum eigenvalue both shows two cointegration vectors in the system. This means the existence of long run association between LE, HE, GDP, Pop and Renewable Energy consumption in China. It suggests that in the long run, health spending and renewable energy consumption determine the life expectancy in China.

**Table 2:** Cointegration results.

**Unrestricted cointegration rank test (Trace)**	
**Hypothesized**		**Trace**	**0.05**	
**No. of CE(s)**	**Eigenvalue**	**Statistic**	**Critical value**	**Prob**.
None	0.423627	160.0189	69.81889	0.0000
At most 1	0.346466	93.89892	47.85613	0.0000
At most 2	0.211132	42.85572	29.79707	0.0009
At most 3	0.109974	14.39696	15.49471	0.0727
At most 4	0.003464	0.416377	3.841466	0.5187
**Unrestricted cointegration rank test (Maximum Eigenvalue)**				
**Hypothesized**		**Max-Eigen**	**0.05**	
**No. of CE(s)**	**Eigenvalue**	**Statistic**	**Critical value**	**Prob**.
None	0.423627	66.12001	33.87687	0.0000
At most 1	0.346466	51.04320	27.58434	0.0000
At most 2	0.211132	28.45876	21.13162	0.0039
At most 3	0.109974	13.98058	14.26460	0.0554
At most 4	0.003464	0.416377	3.841466	0.5187

**Table 3:** VECM results.

**Vectors—dependent variable**	**Wald test**	**ECT (−1)**
LE	2.4287 (0.01)	−2.2230 (0.02)
GDP	−0.0485 (0.96)	−0.0770 (0.00)
HE	2.2341 (0.02)	0.0178 (0.56)
Pop	−4.0923 (0.00)	0.0097 (0.16)
REN	2.5117 (0.01)	−0.0851 (0.00)

After cointegration test, we estimate the error correction model. Wald test estimations present the short run causality, while the lag value of ECT shows the long run causality. The ECM value is negative and significant in first model, where LE is the dependent variable while HE, GDP, POP and REN are independent variables. This implies that health spending improves life expectancy, and an increase in health spending improves life expectancy in China. This suggests the in the long run Chinese government increases life expectancy by increasing health spending. The short run finding also indicates that health spending also improves life expectancy. Similarly, renewable energy also increases life expectancy both in the short run and in the long run, which indicates that renewable energy consumption improves the quality of the environment, thus improving life expectancy both in short run and in the long run. The GDP could also improve life expectancy both in the short run and in the long run, which indicates that a rise in per capita income increases life expectancy. People with good income can access healthcare facilities that improve their health and life expectancy. The population also has long run and short run association with health spending.

Table 4 presents the variance decomposition analysis, which shows a percentage of the error variation in the prediction for all variables. These variations arise from variable's own shocks as well as shocks in other variables within the system. The variance decomposition analysis of LE for the period 1-10 indicates that most variations in LE come from its own shocks. The second major contributor to the variations in LE is the GDP, in the 2^nd^ period, it produces 0.15 variations in LE and gradually increases in the subsequent period, for example, in 10^th^ period, GDP produces 25 percent of the variation in LE. HE variance decomposition findings show that in 2^nd^ period it produces 0.15 percent of variations and gradually increases in the consequent periods for example, in the 10^th^ period, it reaches 6.57 percent of variations. The RE contributes variations in LE and in the 2^nd^ period, it produces 0.38 variations in LE, which gradually increase in the proceeding periods and in the 10^th^ period it produces 8.29 variations in LE. The population produces a lower rate of variation and in 10^th^ period, it only contributes 0.86, which is very low as compared to the other variables in the system.

**Table 4:** Variance decomposition results.

**Period**	**S.E**.	**LE**	**HE**	**GDP**	**POP**	**REN**
**Variance decomposition of LE:**						
1	0.003354	100.0000	0.000000	0.000000	0.000000	0.000000
2	0.010481	99.29877	0.153803	0.156480	0.001570	0.389381
3	0.021763	97.39767	0.548049	0.721901	0.006405	1.325975
4	0.036769	94.34769	1.143442	1.866899	0.016165	2.625803
5	0.054388	90.18574	1.907062	3.754250	0.034498	4.118446
6	0.072976	84.97933	2.804149	6.521738	0.068578	5.626206
7	0.090622	78.85883	3.793914	10.25692	0.132164	6.958171
8	0.105539	72.07752	4.821209	14.93926	0.251062	7.910957
9	0.116564	65.13031	5.798470	20.30493	0.471184	8.295106
10	0.123697	58.92760	6.576715	25.59015	0.863741	8.041796
**Variance decomposition of HE:**						
1	0.003517	0.094399	99.90560	0.000000	0.000000	0.000000
2	0.010374	0.113528	99.75171	0.104487	0.030245	2.58E-05
3	0.020404	0.101481	99.33735	0.443644	0.112956	0.004572
4	0.032800	0.064631	98.58941	1.064288	0.249498	0.032174
5	0.046496	0.032176	97.42878	1.981784	0.443777	0.113487
6	0.060418	0.064305	95.78717	3.163979	0.699667	0.284876
7	0.073678	0.249091	93.63251	4.522609	1.018894	0.576897
8	0.085678	0.677534	91.00602	5.918501	1.398851	0.999094
9	0.096123	1.392152	88.06132	7.187742	1.831232	1.527556
10	0.104945	2.326180	85.07936	8.186555	2.302761	2.105141
**Variance decomposition of GDP:**						
1	0.063563	12.76154	0.029459	87.20900	0.000000	0.000000
2	0.178568	13.89072	0.088352	85.17314	0.007874	0.839915
3	0.332809	14.79481	0.238559	81.58456	0.026361	3.355716
4	0.504172	15.32049	0.552777	76.17222	0.052881	7.901637
5	0.671255	15.22097	1.110641	68.75700	0.086174	14.82521
6	0.820078	14.25890	1.952165	59.54394	0.125511	24.11948
7	0.947251	12.42495	2.996891	49.48057	0.170536	34.92705
8	1.058031	10.21345	3.988237	40.28866	0.222116	45.28753
9	1.159938	8.614869	4.588046	33.67168	0.285134	52.84027
10	1.256014	8.565719	4.624944	30.12083	0.373256	56.31525
**Variance decomposition of POP:**						
1	16,301.62	0.001109	0.210601	0.003662	99.78463	0.000000
2	49,582.68	0.013014	0.097352	0.011925	99.74152	0.136193
3	102,504.5	0.088385	0.030751	0.012695	99.36119	0.506977
4	176,281.2	0.330189	0.015813	0.006551	98.58391	1.063538
5	271,675.8	0.871788	0.050940	0.003837	97.35471	1.718721
6	389,200.2	1.836297	0.126886	0.017090	95.65650	2.363232
7	529,115.5	3.288842	0.229202	0.051757	93.53511	2.895092
8	691,238.9	5.201783	0.341533	0.101448	91.10794	3.247297
9	874,622.9	7.449315	0.448554	0.150872	88.54949	3.401772
10	1,077,233.	9.833781	0.537826	0.184178	86.05899	3.385223
**Variance decomposition of REN:**						
1	0.015642	9.989439	20.92878	0.123099	2.298034	66.66065
2	0.046083	11.65605	21.83227	0.026674	2.612916	63.87209
3	0.092021	12.93121	23.23916	0.033129	2.877553	60.91895
4	0.152433	13.68030	25.19327	0.207769	3.096601	57.82206
5	0.225823	13.77060	27.71603	0.593821	3.267078	54.65248
6	0.310690	13.15041	30.78917	1.186361	3.385197	51.48886
7	0.405793	11.88879	34.33834	1.917495	3.448289	48.40708
8	0.510218	10.17712	38.22693	2.663584	3.456232	45.47613
9	0.623206	8.284853	42.26890	3.278805	3.412526	42.75491
10	0.743825	6.481818	46.26198	3.644308	3.324466	40.28742

**Figure 4:**
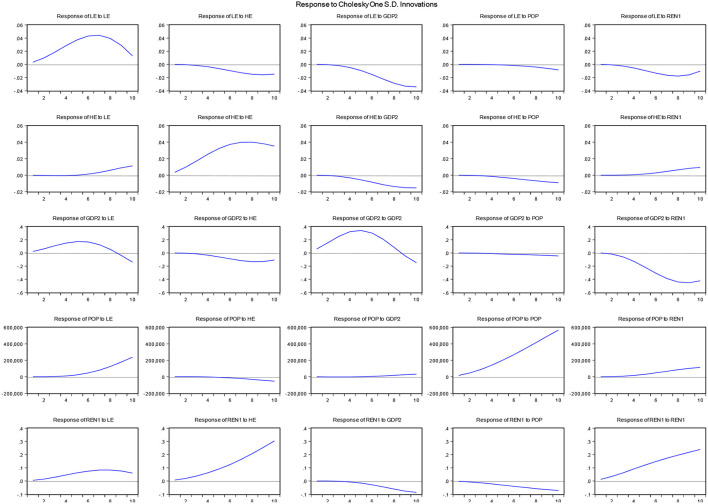
Impulse response results.

Variance decompositions do not provide information regarding the direction of variations in the system, but the impulse response can provide the direction information of the shocks, which is shown in Figure 4. The impulse response function (IRF) illustrates how a change in one variable's standard deviation affects the variations in other variables in the system. It predicts the i^th^ variable innovation (residual) increase by one standard deviation at time t. The graph shows that HE has no effect or minor effect on the LE from 1-5 period; however, from 6^th^ to 10^th^ period HE holds a significant positive impact on LE. And one percent increase in standard deviation in LE has positive and increasing effect on the LE. The variation in GDP shows positive implications for LE from 1^st^ to 8^th^ period; however it become negative in the period of 8^th^-10^th^, this negative association may happen due to higher GDP growth increases environmental pollution mainly to industrialization and economic development, which increases the pollution and reduces LE. Population has positive implications for LE from 4^th^ period to 10^th^ period. The REN shows a positive effect on LE from 2^nd^ period to 10^th^ period. This validates the initial results, and it confirms that renewable energy leads to clean environment and improves life expectancy.

Tables 5, 6 represents the dynamic OLS and Granger Causality Test, which are used as robustness tests. The dynamic OLS model provides regression results regardless of existence of cointegration. The results show the positive and significant implications for the life expectancy. Health spending also has positive and significant implication for the life expectancy, while renewable energy has determine the life expectancy at 10 percent level. The granger causality test estimates bivariate, single variate, or no causality among the variables. The outcomes show that GDP, HE and REN causes LE. Both dynamic OLS and Granger Causality test validate the initial VECM findings. The results of this study support the past studies outcomes such as Kabir (12), Van den Heuvel and Olaroiu (22), Shah et al. (26) and Rodriguez-Alvarez (34).

**Table 5:** Dynamic OLS.

**Variable**	**Coefficient (*P*-values)**
GDP	0.0460 (0.00)
HE	1.0630 (0.01)
POP	1.2708 (0.00)
REN	0.0562 (0.09)
C	54.3264 (0.00)

**Table 6:** Pairwise granger causality tests.

**Null hypothesis**		**F-statistic**	**Prob**.
HE does not Granger Cause GDP		7.07783	0.0013
GDP does not Granger Cause HE		5.90136	0.0036
LE does not Granger Cause GDP		13.6864	5.E-06
GDP does not Granger Cause LE		7.33591	0.0010
POP does not Granger Cause GDP		10.6200	6.E-05
GDP does not Granger Cause POP		20.1974	3.E-08
REN does not Granger Cause GDP		5.94191	0.0035
GDP does not Granger Cause REN		1.54621	0.2173
LE does not Granger Cause HE		0.20139	0.8179
HE does not Granger Cause LE		12.4636	1.E-05
POP does not Granger Cause HE		5.51953	0.0051
HE does not Granger Cause POP		0.45193	0.6375
REN does not Granger Cause HE		1.18033	0.3108
HE does not Granger Cause REN		27.2982	2.E-10
POP does not Granger Cause LE		11.9629	2.E-05
LE does not Granger Cause POP		2.17310	0.1184
REN does not Granger Cause LE		1.71864	0.1838
LE does not Granger Cause REN		13.8051	4.E-06
REN does not Granger Cause POP		34.3257	2.E-12
POP does not Granger Cause REN		11.3992	3.E-05

## Conclusion

The paper analyzes nexus between renewable energy, health spending and life expectancy in China for the period 2000–2020. We applied Vector Error Correction Method for empirical estimations. The life expectancy is used as dependent variable while health spending, GDP per captia, population growth and renewable energy as independent variables. The results of this study have some significant implications for public policy. Our results suggest long run association between health spending, renewable energy, GDP per capita and population. Government can improve life expectancy by increasing health spending in China both in the short run and in the long run. The unidirectional causality reports that government spending causes life expectancy in China both in the short run and in the long run. Therefore, the findings of this study suggest that government should increase health spending in order to improve life expectancy in China. The government may increase healthcare facilities such as setting up new healthcare unites especially in remote and rural areas to make sure that more people have access to the healthcare facilities. In addition, government may provide health insurance to the public and give people easier access to get the healthcare facilities. In addition, renewable energy could have positive implications on life expectancy. The renewable energy consumption has both short-run and long-run association with life expectancy. The consumption of renewable energy could provide clean environment and improve the life expectancy. It suggests that government should raise the production of the renewable energy production and provide incentives to increase renewable energy consumption. Furthermore, Investment in renewable energy consumption should be encouraged and Chinese government should provide subsides interest rate on loan to raise the investment in the renewable energy. The per capita income also improves life expectancy; therefore, economic development is an essential factor for higher life expectancy. Besides, government should make laws for the control of the air pollution to make people turn to renewable energy consumption. Government should provide subsidies on medicine to reduce the pocket heath expenses.

Finally, there are several directions for our study in the future. First, since we used the data for 2000–2020 period in this paper, and it can be expanded by more data with availability in the future. Second, we may use different countries for the data analysis in the subsequent studies. Third, we can consider the social changes and demographic changes in medical care that may affect the health status of the country.

## Data availability statement

The original contributions presented in the study are included in the article/supplementary material, further inquiries can be directed to the corresponding authors.

## Author contributions

Writing—original draft: HL. Review and editing: KZ. Both authors contributed to the article and approved the submitted version.

## Conflict of interest

The authors declare that the research was conducted in the absence of any commercial or financial relationships that could be construed as a potential conflict of interest.

## Publisher's note

All claims expressed in this article are solely those of the authors and do not necessarily represent those of their affiliated organizations, or those of the publisher, the editors and the reviewers. Any product that may be evaluated in this article, or claim that may be made by its manufacturer, is not guaranteed or endorsed by the publisher.
